# Loss-of-Function Mutants and Overexpression Lines of the Arabidopsis Cyclin CYCA1;2/TARDY ASYNCHRONOUS MEIOSIS Exhibit Different Defects in Prophase-I Meiocytes but Produce the Same Meiotic Products

**DOI:** 10.1371/journal.pone.0113348

**Published:** 2014-11-17

**Authors:** Yixing Wang, Ming Yang

**Affiliations:** Department of Botany, Oklahoma State University, Stillwater, Oklahoma, United States of America; Inner Mongolia University, China

## Abstract

In Arabidopsis, loss-of-function mutations in the A-type cyclin *CYCA1;2/TARDY ASYNCHRONOUS MEIOSIS* (*TAM*) gene lead to the production of abnormal meiotic products including triads and dyads. Here we report that overexpression of *TAM* by the *ASK1:TAM* transgene also led to the production of triads and dyads in meiosis, as well as shriveled seeds, in a dominant fashion. However, the partial loss-of-function mutant *tam-1*, an *ASK1:TAM* line, and the wild type differed in dynamic changes in chromosome thread thickness from zygotene to diplotene. We also found that the pericentromeric heterochromatin regions in male meiocytes in *tam-1* and *tam-2* (a null allele) frequently formed a tight cluster at the pachytene and diplotene stages, in contrast to the infrequent occurrences of such clusters in the wild type and the *ASK1:TAM* line. Immunolocalization studies of the chromosome axial component ASY1 revealed that ASY1 was highly expressed at the appropriate male meiotic stages but not localized to the chromosomes in *tam-2*. The level of ASY1, however, was greatly reduced in another *ASK1:TAM* line with much overexpressed *TAM*. Our results indicate that the reduction and increase in the activity of TAM differentially affect chromosomal morphology and the action of ASY1 in prophase I. Based on these results, we propose that either the different meiotic defects or a common defect such as missing ASY1 on the chromosomal axes triggers a hitherto uncharacterized cell cycle checkpoint in the male meiocytes in the *tam* mutants and *ASK1:TAM* lines, leading to the production of the same abnormal meiotic products.

## Introduction

Mitotic cyclins in animals and plants include two groups of homologous and yet different proteins that are termed cyclin As and Bs. In general, the actions of cyclin As occur mostly during the G1, S, G2 phases, and early prophase of the cell cycle, preceding the actions of cyclin Bs that predominantly occur later in the prophase and metaphase [1], [2]. The difference in the timing of action between cyclin As and Bs suggests that cyclin As have functions that are not shared by cyclin Bs. More pronounced cyclin A-specific functions may be anticipated to occur in the prophase I of the meiotic cell cycle since prophase I consists of meiosis-specific substages such as leptotene, zygotene, pachytene and early diplotene prior to the onset of a substage resembling the prophase in the mitotic cell cycle. Although the roles of cyclin As in prophase I in different organisms have been studied, their functions in prophase I are still not well understood.

Cyclin A1 in mice has been reported to play an essential role in male meiosis. Male mice lacking the cyclin A1 protein are defective in desynapsis of homologous chromosomes [3], form congregations of the pericentromeric heterochromatin regions in diplotene spermatocytes, and arrest their cell cycle at late diplotene [4]. Such mutant mice are sterile as the arrested spermatocytes undergo apoptosis. Immunolocalization studies also revealed that cyclin A1 is localized at pericentromeric regions and cyclin A1-dificiency abolishes the chromosomal localization of the passenger protein complex components Survivin and Aurora B, and histone H3 serine 10 phosphorylation [4]. It is also noted that the loss of cyclin A1 cannot be compensated by concurrent expression of cyclin Bs [4]. Cyclin A in Drosophila is also implicated in the regulation of meiotic cell cycle progression by its interaction with the cyclin-dependent kinase (CDK) inhibitor Roughex [5]–[7]. Lack of Roughex induces an extra M phase after meiosis II, and overexpression of Roughex causes a failure to enter meiosis II [5].

In the Arabidopsis *tardy asynchronous meiosis-1* (*tam-1*) mutant that is a partial loss-of-function allele of the A-type cyclin *CYCA1;2*, cell cycle progression is delayed primarily in pachytene and prophase II in male meiosis [8], [9]. In most male meiocytes in the null allele *tam-2*, meiosis I is completed but meiosis II does not occur [10], [11]. However, as found in many plant meiotic mutants [12], [13], there is no cell cycle arrest in the meiocytes and subsequent spores in *tam-2*, which frequently leads to the production of viable unreduced male and female gametes that result in ploidy doubling in the next generation [10]. TAM is predominantly expressed at the pachytene stage in male meiosis but it affects cell cycle progression during and long after the pachytene stage [9], [14]. Genetic studies indicate that TAM acts in the same pathway as OSD1 (also named GIGAS CELL1), TDM1, and SMG7 in meiosis [14], [15]. OSD1 is an inhibitor of APC/C [16] and its mutants have a meiotic phenotype similar to that of the *tam* mutants [11]. SMG7 functions in nonsense-mediated RNA decay and *smg7* mutants are arrested in anaphase II with a high CDKA;1 activity [17]. The *tdm1* mutant undergoes an extra mitosis after meiosis II, which resembles the meiotic defect in *roughex* [18]. TDM1 does not contain a domain with a known function, and how it regulates meiosis at the molecular level is unknown. Epistasis analyses with mutants of these genes have placed *osd1* at the top of the epistatic order, followed by *tdm1*, *smg7*, and *tam* [14], [15].

The above findings collectively show that cyclin As are a major class of cyclins that likely play indispensable roles in meiosis. In the current report, we show that the overexpression of the Arabidopsis A-type cyclin *TAM* by the *ASK1* promoter [19] had a dominant effect that led to the production of the same male meiotic products as in the *tam* loss-of-function mutants. However, upon examination of the chromosome morphology and the subcellular localization of the ASY1 protein in male meiocytes from zygotene to diplotene, different effects of the overexpression and the loss of function of *TAM* were revealed. Our findings thus demonstrate that either the different defects or a common downstream defect derived from these defects can lead to the production of the same abnormal meiotic products.

## Results

### Overexpression of *TAM* has a dominant effect on meiosis and seed development

**Figure 1:**
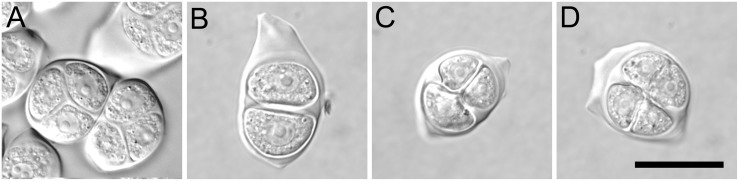
Male meiotic products in the wild type (Col) and T_1_ of *ASK1:TAM2*. (A) Normal tetrads in Col. (B–D) A dyad, a triad, and a tetrad in *ASK1:TAM2*, respectively. Bar = 25 µm.

**Figure 2:**
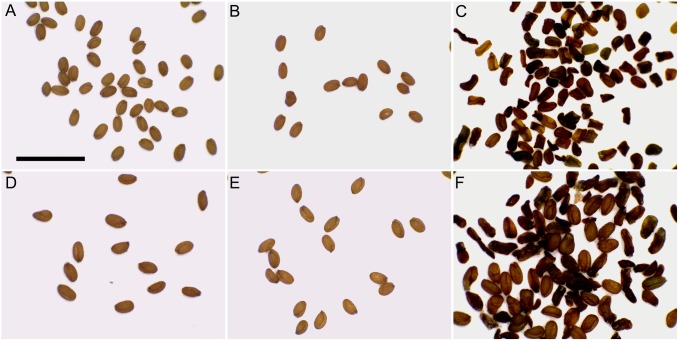
Seeds from Col and T_1_s of *ASK1:TAM*. (A) Diploid Col. (B) Likely diploid normal-looking seeds from an *ASK1:TAM* plant. (C) Likely diploid shriveled and normal-looking seeds from an *ASK1:TAM* plant. Bar = 2 mm. (D) Seeds from tetraploid Col. (E) Likely tetraploid normal-looking seeds from an *ASK1:TAM* plant. (F) Likely tetraploid shriveled and normal-looking seeds from an *ASK1:TAM* plant.

It was previously reported that overexpression of *TAM* by the *ASK1* promoter led to the production of polyploid plants [19]. To investigate the cellular basis for such an effect, a total of 36 T_1_ plants were obtained from six independent transformation experiments that introduced the *ASK1:TAM* transgene into the wild-type plants. Male meiotic products in these plants were then examined. As indicated in Table 1 and Fig. 1, 30 T_1_ plants from four of the transformation experiments produced either apparently normal tetrads or abnormal products such as dyads or a mixture of dyads, triads, and tetrads. Four T_1_ plants from one remaining transformation experiment produced only tetrads while two plants from still another transformation experiment produced only dyads or a mixture of dyads, triads, and tetrads. The abnormal meiotic products should also have occurred in female meiosis in some of these lines because progeny from at least one of the lines, *ASK1:TAM1*, were tetraploid [19]. Furthermore, 34 of these plants produced variable amounts of dark and collapsed seeds (Fig. 2). The number of lines producing the abnormal seeds is much larger than that of the lines producing the abnormal meiotic products, indicating that the seed defect is independent of the production of abnormal meiotic products, and that seed development is more sensitive to the overexpression of *TAM* than meiotic cell cycle progression. The variation in the severity of these defects likely resulted from the variation in the expression level of the transgene in these plants [19]. Because heterozygosity for the transgene is expected in T_1_ plants, the presence of these abnormalities in the T_1_ plants indicates that the transgene had a dominate effect on meiosis and seed development.

**Table 1:** Male meiotic products in *ASK1:TAM* at the T_1_ generation.

Experiment	Number of T_1_s	Meiotic products
1	9	Tetrads
	5	Dyads
2	6	Tetrads
	3	Dyads
	1	Mixture of dyads, triads, and tetrads
3	4	Tetrads
4	2	Tetrads
	2	Dyads
5	1	Tetrads
	1	Dyads
6	1	Dyads
	1	Mixture of dyads, triads, and tetrads

### Differential effects of *ASK1:TAM* and *tam-1* on chromosomal thickness in zygotene-to-diplotene male meiocytes

**Figure 3:**
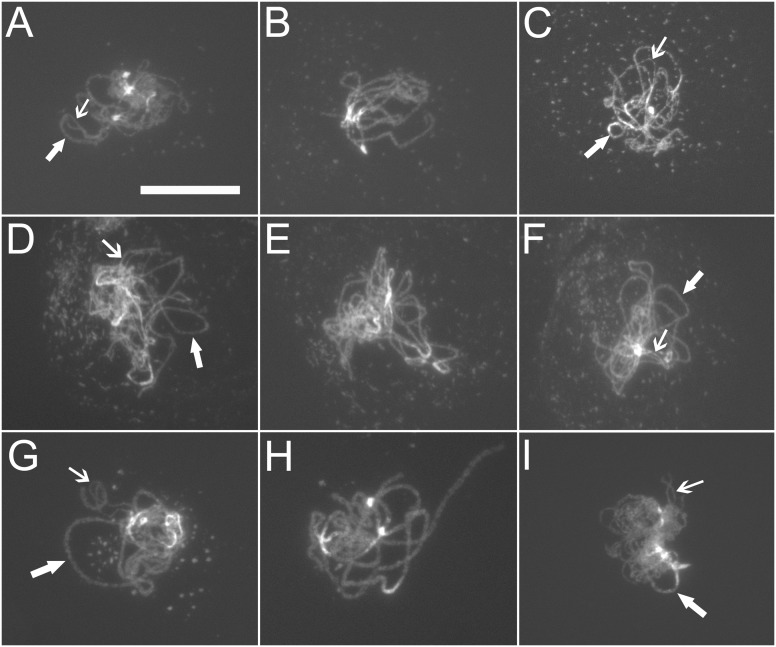
Morphology of spread chromosomes. (A–C) Zygotene, pachytene, and early diplotene chromosomes in Col, respectively. (D–F) Zygotene, pachytene, and early diplotene chromosomes in *tam-1*, respectively. (G–I) Zygotene, pachytene, and early diplotene chromosomes in *ASK1:TAM2*, respectively. Thin arrows, unsynapsed zygotene or desynapsed diplotene chromosome regions. Thick arrows, synapsed chromosome regions. Bar = 25 µm.

**Figure 4:**
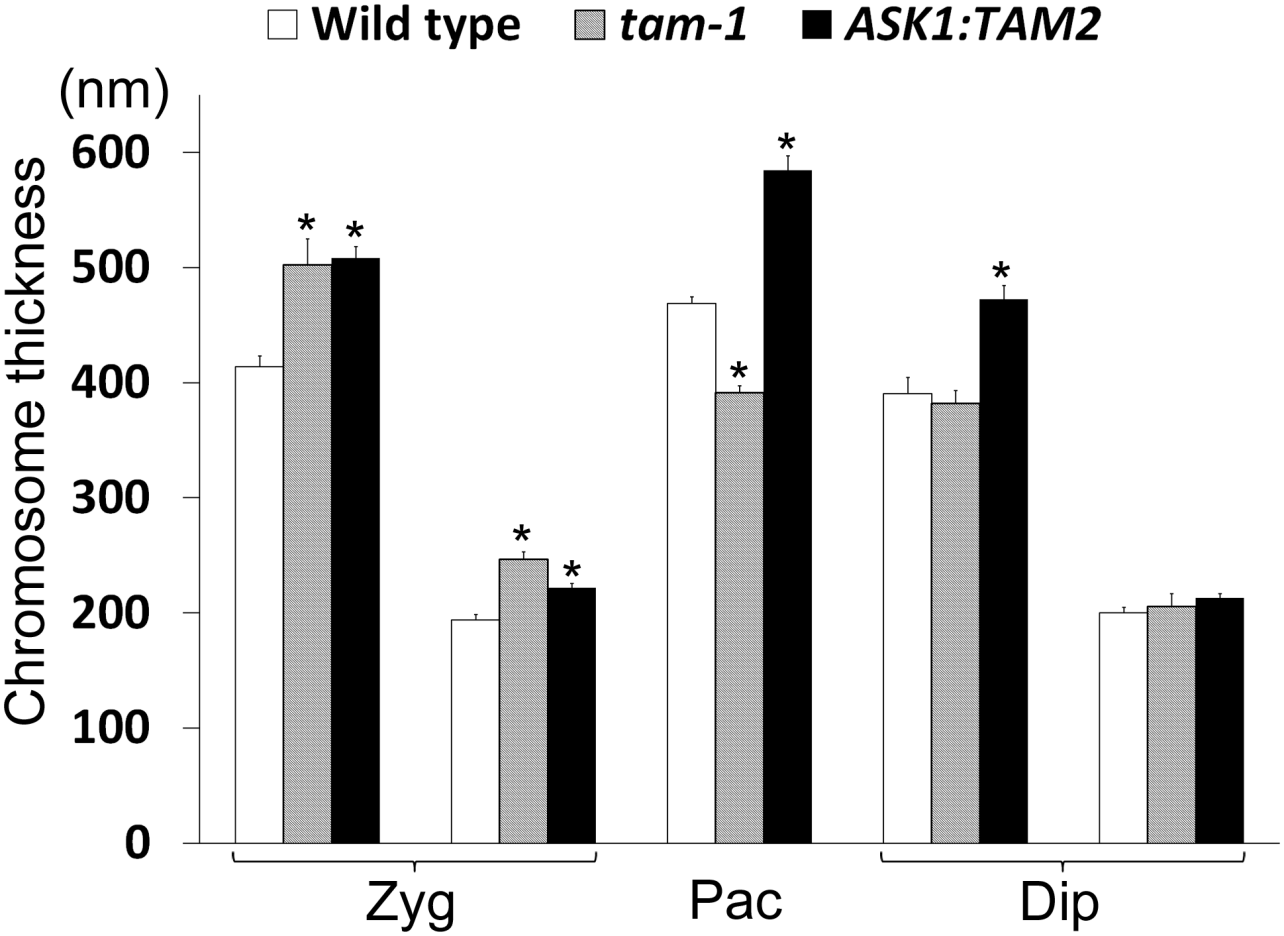
Thicknesses of synapsed, unsynapsed, and desynapsed chromosomes in male meiocytes in Col, *tam-1*, and *ASK1:TAM2*. Shown are averages ± standard errors. *The values are statistically different from the corresponding values of Col. All statistical differences are based on t-test with p<0.01 and n≥10.

Because *ASK1:TAM* plants produced male meiotic products similar to those in *tam-1* and *tam-2*, we next investigated spread chromosomes from male meiocytes in an attempt to detect similarities or dissimilarities among the wild type, *tam-1*, *ASK1:TAM2*. *tam-1* was chosen because it has a mild meiotic defect so that a population of homozygous and diploid seeds is available [8]; a population of homozygous *tam-2* seeds is available only as polyploid seeds [10]. The *ASK1:TAM2* line used in this investigation has been shown to have a moderate increase in the amount of the *TAM* transcript and was confirmed to be diploid [19]. No apparent defect in chromosome pairing and synapsis was observed throughout prophase I in the three genotypes, however, subtle differences in the thickness of the chromosome thread appeared to exist among them. In particular, synapsed chromosome regions of either thicker or thinner than those of the wild type appeared to exist in *tam-1* (Fig. 3A–F), while the synapsed chromosome regions often appeared thicker in *ASK1:TAM2* than in the wild type (Fig. 3G–I). To quantify such differences with respect to the cell cycle stages, the thicknesses of unsynapsed, synapsed, and desynapsed chromosome regions were measured from zygotene to early diplotene, and the results are shown in Fig. 4. In zygotene, the average thicknesses of unsynapsed and synapsed chromosome threads in the wild type were smaller than the corresponding thicknesses in *tam-1* and *ASK1:TAM2*. In pachytene, the average thickness of the synapsed chromosome threads in the wild type was larger than that in *tam-1* but smaller than that in *ASK1:TAM2*. In diplotene when both synapsed and desynapsed chromosomes were present in a single meiocyte, the average thickness of the desynapsed chromosome threads in the wild type was not statistically different from that in *tam-1* and *ASK1:TAM1*. However, the average thickness of synapsed chromosome threads in the same diplotene meiocytes in the wild type was smaller than that in *ASK1:TAM2* but not statistically different from that in *tam-1*. Fig. 4 also shows that during cell cycle progression from zygotene to diplotene, within the wild type, the synapsed chromosome threads increased the average thickness in pachytene when compared to that in zygotene and decreased the average thickness in diplotene to a value similar to that in zygotene. The unsynapsed chromosome threads in zygotene and the desynapsed chromosome threads in diplotene in the wild type were about the same thickness on average. The same dynamics in the average thickness of chromosome threads also occurred in *ASK1:TAM2*. However, different dynamics occurred in *tam-1*; on average, both synapsed chromosome threads in pachytene and diplotene and desynapsed chromosome threads in diplotene were thinner than those of the synapsed and unsynapsed chromosome threads in zygotene, respectively. These results indicate that the loss of function and overexpression of *TAM* have different effects on chromosomal morphogenesis from zygotene to diplotene.

### Pericentromeric heterochromatin regions tend to cluster in *tam-1* and *tam-2*

**Figure 5:**
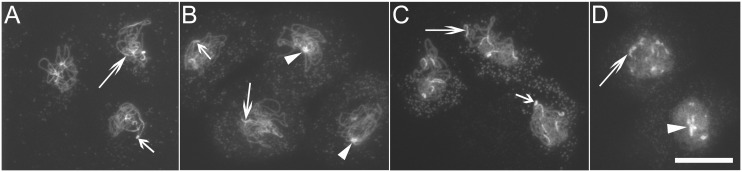
Spread chromosomes at the pachytene or diplotene stage showing scattered and clustered pericentromeric heterochromatin regions. (A) Col. (B) *tam-1*. (C) *ASK1:TAM2*. (D) *tam-2*. Bar = 25 µm.

Pericentromeric heterochromatin regions in cyclin A1-deficient male mice congregate in spermatocytes at a late diplotene stage [4]. We also investigated spatial distribution patterns of pericentromeric heterochromatin regions in male meiocytes at the pachytene or diplotene stage in the WT, *tam-1*, *tam-2* (octaploid), and *ASK1:TAM2*. Both cells with a tight cluster of pericentromeric heterochromatin regions and cells without such a cluster were found in all the genotypes investigated. Morphologically, cells without such a cluster were characterized by the presence of scattered and elongated pericentromeric heterochromatin regions (Fig. 5, long arrows) that were frequently accompanied by clearly visible joined ends of chromosomes 2 and 4 harboring the rDNA regions (Fig. 5, short arrows) [20]. In contrast, cells containing clustered pericentromeric heterochromatin regions had a single large DAPI-staining-intense area (Fig. 5B, arrow heads) without the appearance of the scattered and elongated pericentromeric heterochromatin regions and with hardly visible the joined ends of chromosomes 2 and 4. These morphological differences were used to score the cells of such. In plants grown at 22°C, formation of a tight cluster of the pericentromeric heterochromatin regions was found only in 6% of male meiocytes in both the wild type (n = 53) and *ASK1:TAM2* (n = 85), but it was 23% in *tam-1* (n = 96) and 41% in *tam-2* (n = 27). Because the severity of the *tam-1* meiotic defect was sensitive to elevated temperature [8], pachytene or diplotene male meiocytes with or without the cluster of the pericentromeric heterochromatin regions were also investigated in wild-type and *tam-1* plants grown at 28°C. At the elevated temperature, the percentage of cells containing the cluster was still 6% in the wild type (n = 122) whereas it was 40% in *tam-1*, consistent with *tam-1* being a temperature-sensitive mutant. Taken together, our results indicate that *tam-1* and *tam-2* differ from the wild type and *ASK1:TAM2* in the spatial patterning of the pericentromeric heterochromatin regions in mid-to-late prophase-I male meiocytes.

### Chromosomal loading and abundance of ASY1 are differentially affected in *tam* mutants and *ASK1:TAM* lines

**Figure 6:**
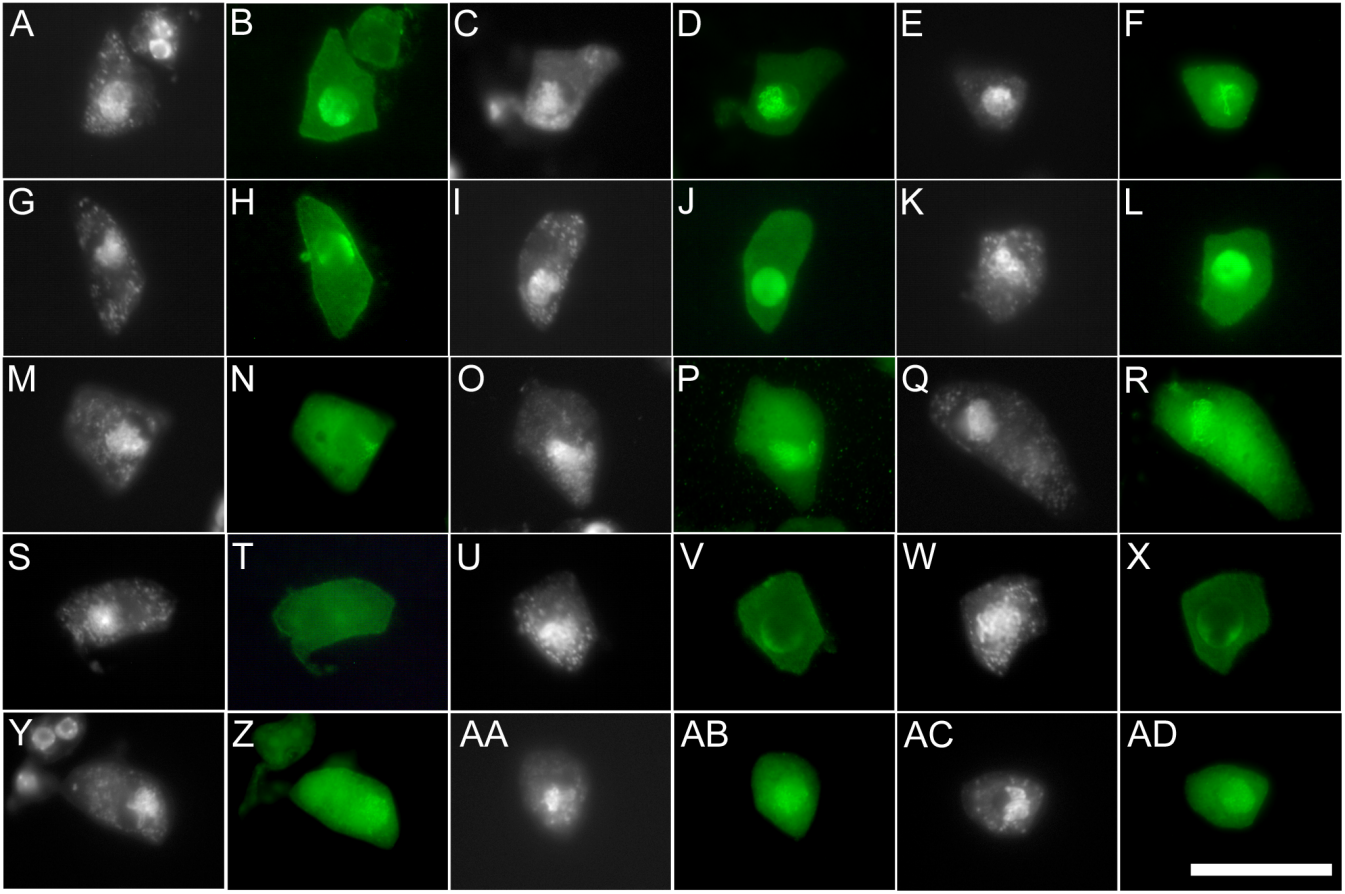
Immunolocalization of ASY1 in male meiocytes. Shown are DAPI and corresponding anti-ASY1 (green fluorescence) images of three stages (starting from left as the earliest) for each genotype. (A–F) Leptotene, zygotene, and pachytene stages in Col. (G–L) Leptotene, zygotene, and pachytene stages in diploid *tam-2*. (M–R) Leptotene or early zygotene, zygotene, and pachytene stages in *tam-1*. (S–X) Leptotene, zygotene, and pachytene stages in *ASK1:TAM3*. (Y–AD) Leptotene or early zygotene, zygotene, and pachytene stages in *ASK1:TAM2*. Bar = 25 µm.

To further uncover how the *tam* mutations and overexpression of *TAM* affect prophase I, we investigated the expression and subcellular localization of the ASY1 protein in male meiocytes at the stages from leptotene to pachytene by immunolocalization using an antibody against Arabidopsis ASY1 [21]. It was reported that ASY1, a protein with a HORMA domain in the N-terminus, progressively associates with the axes of meiotic chromosomes from leptotene to pachytene [21], which can be used as a marker for prophase-I progression. The plant lines used in this investigation included the wild type, *tam-1*, *tam-2*, *ASK1:TAM2*, and *ASK1:TAM3*. Here *tam-2* was diploid that was identified from the progeny of a plant heterozygous for *tam-2*, and *ASK1:TAM3* was also diploid with a much higher *TAM* expression than *ASK1:TAM2* [19]. In the wild type, ASY1 started to appear in the nuclei in leptotene meiocytes and only a portion of the protein potentially colocalized with the chromatin (Fig. 6A and B). The late zygotene meiocytes in the wild type showed strong colocalization of ASY1 and the chromatin with the chromosome threads clearly visible with the green fluorescence (Fig. 6C and D). The wild type meiocytes at the pachytene stage still showed ASY1 and chromatin colocalization but the signal seemed diminishing (Fig. 6E and F). This temporal pattern of ASY1 colocalization with the chromatin in intact meiocytes largely conforms to what is previously found with spread chromosomes, but in the intact meiocytes, it was clear that from leptotene to pachytene there was always a portion of ASY1 that did not associate with the chromatin in the nucleus. The timing of expression and nuclear localization of ASY1 in the meiocytes were not affected in *tam-2*, but its colocalization with the chromatin was disrupted; the fluorescence signal was smooth throughout the nucleus, indicating that there was not such colocalization or at least the vast majority of the ASY1 molecules remained unassociated with the chromatin (Fig. 6G–L). There was no obvious difference in the timing of expression and nuclear localization pattern of ASY1 between the wild type and *tam-1* (Fig. 6M–R), consistent with *tam-1* having a mild meiotic defect comparing to the null allele *tam-2*. In *ASK1:TAM3*, no apparent signal of ASY1 was detected in leptotene meiocytes (Fig. 6S and T), and only weak signals of ASY1 could be observed in the nuclear peripheries in zygotene and pachytene meiocytes (Fig. 6U–X). These signals did not appear to colocalize with at least the main bodies of the chromosome threads. The signals of ASY1 in *ASK1:TAM2* appeared similar to those in the wild type in terms of the timing of expression and distribution pattern in the nuclei (Fig. 6Y–AD), but the overall signal level appeared lower than those in the wild type, *tam-1*, and *tam-2*. The mild effect of *ASK1:TAM2* on ASY1 abundance is in contrast to the severe effect of *ASK1:TAM3* on ASY1 abundance and distribution in the nucleus, which is consistent with *ASK1:TAM3* having a much higher expression of *TAM* than *ASK1:TAM2* [19]. Taken together, our results indicate that, during synapsis, TAM normally promotes the localization of ASY1 to the chromatin while overexpression of *TAM* reduces the amount of ASY1 in the nucleus. In other words, the same consequence of lack of ASY1 loading onto the chromatin, though by different mechanisms, can occur in both the *tam* mutants and *ASK1:TAM* lines.

## Discussion

### The possible mechanism for producing the same meiotic products in the *tam* mutants and *ASK1:TAM* lines

The recombination checkpoint is known to regulate the progression of prophase I in yeast [22] and metazoans [23], [24]. Recently, the concept of meiotic checkpoint network (MCN) was proposed to better describe the highly connected network of a relatively small number of proteins involved in the checkpoint regulation of recombination and homologous chromosome synapsis [25]. While the MCN regulates the normal progression of prophase I, a defect in recombination or homologous chromosome synapsis activates the MCN that typically leads to cell death in metazoans or cell cycle arrest in yeast [25]. However, cell cycle arrest or cell death is conspicuously absent in plant meiotic mutants that are defective in recombination and/or synapsis in prophase I, suggesting that either plants lack such a checkpoint mechanism or the MCN in plants is much relaxed comparing to the MCN in yeast and metazoans [26]. Findings from the current and previous investigations demonstrate that the *tam* mutants and *ASK1:TAM* lines have different meiotic defects in prophase I and yet they produce the same abnormal meiotic products, namely triads and dyads. This observation suggests that a common cell cycle checkpoint may be triggered in prophase I in the *tam* mutants and the *ASK1:TAM* lines. Activation of the checkpoint likely delays cell cycle progression as previously demonstrated by the effect of *tam-1* on the durations of cell cycle phases, leading to the production of triads and dyads [8]. The checkpoint may be triggered by the different defects in the *tam* mutants and the *ASK1:TAM* lines, or alternatively, by a subsequent common defect derived from the different defects, such as the missing or reduction of ASY1 on meiotic chromosomal axes in both the *tam* mutants and the *ASK1:TAM* lines. The latter scenario can be envisioned to occur with either the defect in loading ASY1 onto the chromosomes in the *tam* mutants or the reduction of ASY1 in the nuclei of meiocytes in the *ASK1:TAM* lines (Fig. 6). In principle, the MCN can be activated at multiple nodes of the network. It is recognized that the results from this investigation can be explained by the activation of the MCN in Arabidopsis by either the same defect at the same node or different defects at separate nodes of the MCN, which leads to a delay in cell cycle progression.

Currently there is no knowledge about the components of the MCN in plants. Because OSD1, TDM1, SMG7, and TAM are in the same pathway in the regulation of meiotic cell cycle progression, it may be speculated that OSD1, TDM1, SMG7, and TAM are either components of the MCN or linked to components of the MCN. It may also be speculated that adequate loading of ASY1 onto chromosomal axes may serve as a signal for deactivating the MCN.

### Loading of ASY1 onto chromatin is not required for synapsis

Loss of ASY1 causes a severe defect in synapsis of homologous chromosomes (the protein was named after this defect) and the fertility of the *asy1* mutant is low [18], [21]. In the *tam* mutants, ASY1 is present in the nuclei of meiocytes but loading of ASY1 onto the chromosomes is impaired, and yet the homologous chromosomes can undergo synapsis [9], [10]. These findings together suggest that homologous chromosome synapsis does not require the chromosomal loading of ASY1 but the presence of ASY1 in the nucleus. Normal cell cycle progression during synapsis, on the other hand, does require the chromosomal loading of ASY1. Hop1p in yeast is moderately homologous (24% identities, 45% positives, and 19% gaps in approximately the N-terminal halves of the proteins) to ASY1. Hop1p interacts with two other yeast meiosis-specific proteins Red1p and Mek1p and is involved in both recombination and recombination checkpoint control [27], [28]. The newly proposed MCN in yeast and metazoans includes HORMA-domain proteins [25]. How the meiotic function of ASY1 in Arabidopsis is compared with those of HORMA-domain proteins in yeast and metazoans remains to be investigated.

### TAM1 may be involved in the regulation of chromatin state

In principle, chromosome size may be a function of chromatin state that is largely determined by the modifications of histones and other chromosome-associated proteins. Phosphorylation is one of the widely occurred modifications on these proteins. The unusually thin and thick chromosome threads found in *tam-1* and *ASK1:TAM2* thus may reflect altered chromatin states due to the decreased and increased TAM-CDK activity in these plants, respectively. Another hint for the involvement of TAM in the regulation of chromatin state is the tendency for the pericentromeric heterochromatin regions to form a cluster in *tam-1* and *tam-2*, which is similar to the congregation of pericentromeric heterochromatin regions in diplotene in cyclin A1-deficent male mice [4]. The congregation of pericentromeric heterochromatin regions in mice presumably results from the absence of cyclin A1-dependent localization of Aurora B at the pericentromeric regions, which in turn prevents the phosphorylation of serine 10 on histone H3 at these regions [4]. Lack of H3 histone serine 10 phosphorylation may further affect the chromatin state by withholding the component of heterochromatin, Heterochromatin Protein 1, at heterochromatin [29]. It is possible that such regulatory mechanism also exits in Arabidopsis and involves TAM.

### TAM plays distinct roles in meiocytes and the seed

*TAM* is normally expressed in the seed although its function in the seed is uncharacterized [19]. In this investigation, we found that *ASK1:TAM* T_1_ plants produced shriveled seeds even when they produced normal male meiotic products. This observation argues that either TAM plays a different role in the seed than in the meiocytes or the seed is more sensitive to the overexpression of *TAM* than the meiocytes. However, another intriguing observation is that triploid or tetraploid seeds develop normally in diploid *tam-2* [10], [11], indicating that the loss of function of *TAM* leads to an outcome different from that of *TAM* overexpression. The different outcomes in seed development resulting from the *tam-2* mutation and *TAM* overexpression, in contrast to the same outcome in meiosis, seem to support the notion that TAM plays a different role in the seed than in the meiocytes. Recently *TAM* was found to have a subtle effect on nuclear size in differentiated cells in Arabidopsis vegetative tissues, and the same *tam mutants* and *ASK1:TAM* lines exhibited similar nuclear size increases [19], reminiscent of the same meiotic products produced in these plants. How these seemingly different but presumably related phenomena are reconciled at the molecular level awaits future investigations.

## Materials and Methods

### Plant materials and growth conditions

The plants used in this investigation are of the Columbia accession of *Arabidopsis thaliana*. The plants were grown at 22°C with 16h light/8h dark in growth chambers or a growth room on an artificial soil (Sunshine MVP, Sun Gro Horticulture, Seba Beach, Canada). The *tam* mutants and *ASK1:TAM1-3* lines have been previously described [19]. In particular, *ASK1:TAM1* is tetraploid and *ASK1:TAM2* and *ASK1:TAM3* are diploid, and their levels of the *TAM* transcript were approximately 1.7, 2, and 119 folds of the wild-type level, respectively [19]. All the transgenic plants used in this investigation are presumed to be homozygous for the transgene because the seeds of each line were 100% resistant to the transgene selecting chemical gentamicin.

### Light microscopy

Fixed and fresh whole-mount male meiocytes and meiotic products were examined and scored with bright-field, differential interference contrast, and fluorescence microscopy as previously described [8], [30]. Seed phenotypes were examined under a Leica S6D dissecting microscope and photographed using a Leica EC3 digital camera on the same microscope. Chromosome spread samples were prepared and investigated as previously described [31]. The meiotic stages were determined according to multiple factors, including the chromosome morphology, distribution of DAPI-stained organelles in the cell–asymmetrical and symmetrical distributions around the nucleus coincide with zygotene and post-zygotene stages, respectively [32], the location of the nucleus–peripheral and central localizations also indicate zygotene and post-zygotene stages, respectively, the range of stages in the same anther, in the medial and lateral anthers of the same bud, and in consecutive buds [8], and the percentage of binucleate tapetal cells accompanying the meiocytes [31]. Sample preparation for immunolocalization of ASY1 was conducted as previously described [33]. The polyclonal antibody against ASY1 was used after a 1∶500 dilution with a PBS buffer also containing 0.1% Triton X-100 and 1% BSA. All the figures were assembled, and their brightness and contrast moderately adjusted, in Adobe Photoshop CS2.

### Measurement of chromosome thickness

Chromosome thicknesses were measured with SigmaScan Pro 5. For each cell, three measurements on separate chromosomes or separate regions of the same chromosome were conducted and their average was used to represent the thickness of the synapsed, unsynapsed, or desynapsed chromosomes in that cell. The averages presented in the Results are based on measurements from multiple cells.
